# Metagenomic insights into zooplankton‐associated bacterial communities

**DOI:** 10.1111/1462-2920.13944

**Published:** 2017-10-27

**Authors:** Daniele De Corte, Abhishek Srivastava, Marja Koski, Juan Antonio L. Garcia, Yoshihiro Takaki, Taichi Yokokawa, Takuro Nunoura, Nathalie H. Elisabeth, Eva Sintes, Gerhard J. Herndl

**Affiliations:** ^1^ Department of Limnology and Oceanography Center of Functional Ecology University of Vienna, Althanstrasse 14 1090 Vienna Austria; ^2^ National Institute for Aquatic Resources, Section for Oceans and Arctic Technical University of Denmark, Kavalergaarden 6 2920 Charlottenlund Denmark; ^3^ Department of Subsurface Geobiological Analysis and Research Japan Agency for Marine‐Earth Science and Technology (JAMSTEC), Natushima 2‐15 Yokosuka Kanagawa 237‐0061 Japan; ^4^ Department of Marine Microbiology and Biogeochemistry Royal Netherlands Institute for Sea Research Utrecht University, PO Box 59 AB Den Burg 1790 The Netherlands; ^5^ Present address: Research and Development Center for Marine Biosciences, Japan Agency for Marine‐Earth Science and Technology (JAMSTEC), Natushima 2‐15 Yokosuka Kanagawa 237‐0061 Japan

## Abstract

Zooplankton and microbes play a key role in the ocean's biological cycles by releasing and consuming copious amounts of particulate and dissolved organic matter. Additionally, zooplankton provide a complex microhabitat rich in organic and inorganic nutrients in which bacteria thrive. In this study, we assessed the phylogenetic composition and metabolic potential of microbial communities associated with crustacean zooplankton species collected in the North Atlantic. Using Illumina sequencing of the 16S rRNA gene, we found significant differences between the microbial communities associated with zooplankton and those inhabiting the surrounding seawater. Metagenomic analysis of the zooplankton‐associated microbial community revealed a highly specialized bacterial community able to exploit zooplankton as microhabitat and thus, mediating biogeochemical processes generally underrepresented in the open ocean. The zooplankton‐associated bacterial community is able to colonize the zooplankton's internal and external surfaces using a large set of adhesion mechanisms and to metabolize complex organic compounds released or exuded by the zooplankton such as chitin, taurine and other complex molecules. Moreover, the high number of genes involved in iron and phosphorus metabolisms in the zooplankton‐associated microbiome suggests that this zooplankton‐associated bacterial community mediates specific biogeochemical processes (through the proliferation of specific taxa) that are generally underrepresented in the ambient waters.

## Introduction

Zooplankton and microbes are fundamental components of the ocean's lower food web. Crustacean zooplankton release copious amounts of particulate organic matter (POM) originating from phytoplankton, heterotrophic microzooplankton and detritus into the ambient water (Heinle *et al*., 1977; Calbet, 2001). Heterotrophic microbes are responsible for most of the dissolved organic matter (DOM) mineralization in the open ocean (Azam *et al*., 1983; Cherrier *et al*., 1996). These two components of the marine food web are generally treated as separate entities only connected through the trophic cascades, albeit, microbes and zooplankton are dynamically linked at different ecological levels (Azam and Malfatti, 2007; Tang *et al*., 2010). For example, microbes may exploit zooplankton as a nutrient‐ and carbon‐enriched microhabitat by colonizing its exoskeleton and/or gut (Carman and Dobbs, 1997; Tang *et al*., 2010). Additionally to the nutrient‐enriched conditions, the zooplankton's gut provides a hypoxic environment that may facilitate marginal but important anaerobic processes such as denitrification, dissimilatory nitrate or nitrite reduction and methanogenesis in the oxygenated open waters (De Angelis and Lee, 1994; Tang *et al*., 2011; Glud *et al*., 2015; Stief *et al*., 2017). Finally, the zooplankton's acidic digestive tract may promote iron recycling and solubilization via multiple pathways involving microbes (Tang *et al*., 2011; Nuester *et al*., 2014; Schmidt *et al*., 2016). These processes deliver bioavailable iron to the ambient water that can be utilized by phytoplankton, thus promoting iron fertilization (Schmidt *et al*., 2016).

Even though the majority of microbes are free‐living, several studies have shown that the abundance of zooplankton‐associated bacteria can be orders of magnitude higher than free‐living bacteria on a per volume base (Tang *et al*., 2006; Tang *et al*., 2010; Tang *et al*., 2011; Schmidt *et al*., 2016). Culture‐based studies indicated that zooplankton‐associated microbial (i.e., bacterial and archaeal) communities consist of similar taxa as ambient water communities (Delille and Razouls, 1994; Hansen and Bech, 1996). However, recent culture‐independent studies reveal a strong niche partitioning of bacterial communities between the zooplankton and the surrounding waters probably driven by the different physico‐chemical conditions (Grossart *et al*., 2009; De Corte *et al*., 2014). These findings also suggest an active microbial exchange between the two habitats in which each environment favours the proliferation of specific taxa generally underrepresented in the other environment (Grossart *et al*., 2009; De Corte *et al*., 2014).

The aim of this study was to compare the phylogenetic composition of the bacterial community inhabiting the ambient water with that associated with different crustacean zooplankton species collected in the North Atlantic Ocean using 16S rRNA gene Illumina sequencing. Zooplankton individuals were collected during day and night to assess whether the feeding status influences the zooplankton‐associated bacterial community. Finally, metagenomic analyses provided insights into the yet underexplored metabolic interaction between the zooplankton and the associated bacterial community.

## Results

### Bacterial community richness and diversity

**Figure 1:**
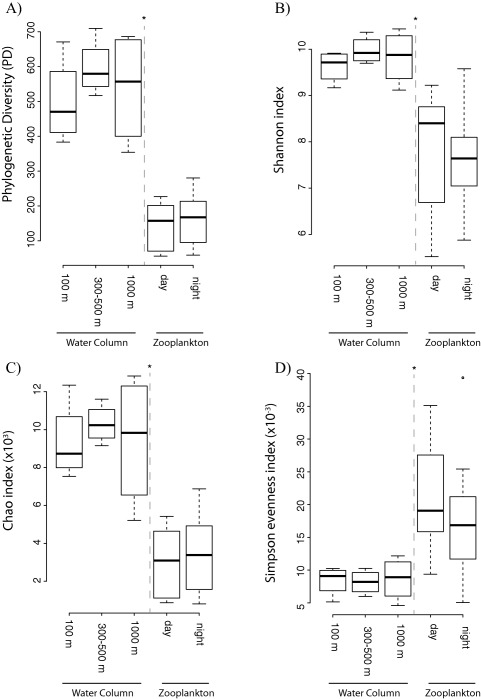
Box plot of data of phylogenetic (A) and Shannon (B) diversity indexes, Chao species richness (C) and Simpson evenness index (D) obtained from ambient water (collected at three different depths layers) and zooplankton‐associated (sampled during day and night) bacterial communities. The bottom and the top of the box represent the first and the third quartiles, while the thick horizontal line represents the median.

Rarefaction analyses [phylogenetic diversity (PD), Chao richness and observed operational taxonomic units (OTUs)] showed clear differences between zooplankton‐associated and ambient water bacterial communities (Supporting Information Fig. S1). The rarefaction curves for zooplankton‐associated bacterial communities approached a plateau, however, the rarefaction curves of the ambient water communities did not level off (Supporting Information Fig. S1). Additionally, the PD and Chao richness were significantly higher (T‐test, P < 0.001) for the ambient water than the zooplankton‐associated bacterial community (Fig. 1A–C, Supporting Information Table S1). Furthermore, the Simpson evenness was significantly higher in the zooplankton‐associated than in the free‐living bacterial community (Fig. 1D, Supporting Information Table S1). No significant differences were found between diversity, richness and evenness indexes of ambient water bacterial communities collected at different depth layers or between zooplankton–associated communities collected during the day versus night (ANOVA on rank, P > 0.001) (Fig. 1 and Supporting Information Fig. S1, Table S1). Members of the zooplankton‐associated bacterial community were more evenly distributed than those of the ambient water and exhibited a comparatively low diversity with few but abundant OTUs.

### Bacterial community composition in zooplankton versus ambient water

**Figure 2:**
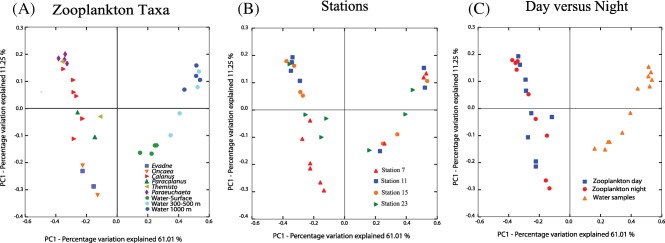
Principal coordinates analysis (PCoA) of zooplankton‐associated and ambient water bacterial communities from individual samples. Bacterial communities isolated from a specific zooplankton group and depth in the water column (A), station (B) or time of the day (C) are represented by the different symbols.

Principal Coordinates Analysis (PCoA) using weighted Unifrac distances (Lozupone *et al*., 2011), was used to statistically explore and visualize the similarity between the different bacterial communities. PCoA analysis clearly separated ambient water and zooplankton‐associated bacterial communities (Fig. 2), with the first coordinate accounting for 61% and the second for 11% of the variance. Zooplankton‐associated communities clustered together and, within them, clustered according to the taxon of the zooplankton individuals (Fig. 2A) and to the sample location (Fig. 2B). We did not observe clustering associated to the time of collecting (day vs. night) the zooplankton (Fig. 2C). The shared OTUs among groups of samples (surface and mesopelagic free‐living bacterial communities and zooplankton‐associated bacterial communities collected during day and night) were determined with Mothur (Schloss *et al*., 2009) from the OTU distribution obtained in QIIME. Only 0.7% of the OTUs were shared and ubiquitously present in the zooplankton‐associated and ambient water communities (Supporting Information Fig. S2). The zooplankton samples (day vs. night) shared only ∼10% of the total OTUs, whereas the communities from the two depth layers (subsurface and mesopelagic) shared ∼18% of the OTUs. Therefore, the number of shared OTUs was higher within ambient water bacterial communities than within zooplankton‐associated bacterial communities. In addition, the contribution of unique OTUs was higher in the ambient water than in zooplankton‐associated bacterial communities (Supporting Information Fig. S2).

### Taxonomic characterization of the bacterial communities

**Figure 3:**
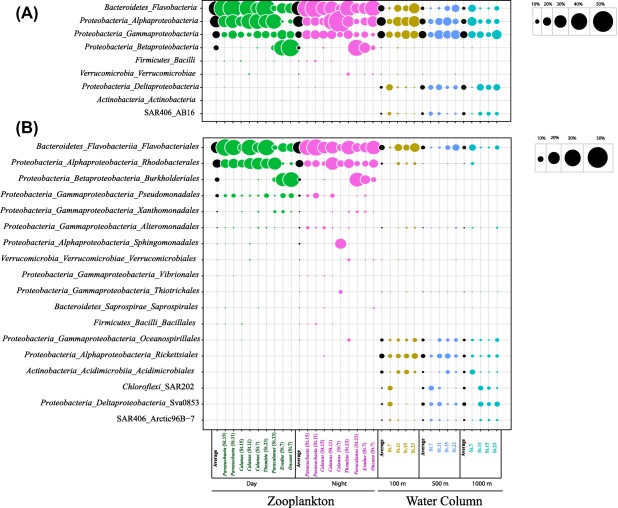
Relative contribution of the more abundant phylogenetic classes (A) and orders (B) to the total number of 16S rDNA sequences obtained from zooplankton‐associated bacterial communities sampled during day and night, and from ambient water bacterial communities collected at three depths (100 m, 500 m and 1000 m).

The phylogenetic analysis of the 16S rRNA gene sequences performed in QIIME using the Greengenes database revealed the dominance of three bacterial phyla in ambient water communities [*Proteobacteria*, *Bacteroidetes* and Marinimicrobia (SAR406)] and of two bacterial phyla (Proteobacteria and *Bacteroidetes*) in the zooplankton‐associated communities (Fig. 3A). At the family level (Fig. 3B), *Flavobacteriales* and *Rhodobacterales* dominated the zooplankton‐associated communities (32.3% ± 11.8% and 33.7% ± 8.2% respectively), followed by *Burkholderiales* and *Pseudoalteromonadales* (ranging between 2% and 10% of the total community). There were no significant differences observed between day and night samples (Fig. 3, Table 1). In the ambient water, the bacterial community consisted of *Flavobacteriales* (11%), *Rickettsiales* (10%), *Deltaproteobacteria* (7%), *Oceanospirillales* (7%) and *Acidimicrobiales* (6%), followed by *Chloroflexi* (SAR202) (5%), *Marinimicrobia* (SAR406) (4%), *Rhodobacteriales* (3%) and *Alteromonadales* (3%) (Fig. 3B, Table 1). Three taxa (*Flavobacteriales*, *Rhodobacterales, Alteromonadales*) contributed significantly to both the ambient water and zooplankton‐associated bacterial communities; however, their relative contribution to the respective bacterial community differed between the two environments (Fig. 3, Table 1). Conversely, some phylogenetic groups, such as *Rickettsiales* (SAR11), *Acidimicrobiales*, SAR202, Sva0853 and Arctic96B‐7 were specific for the ambient water (Fig. 3, Table 1).

**Table 1:** Relative contribution (%, SD) of the most abundant family to the total number of sequences associated with zooplankton samples collected during day (750 m) and night (250 m) and from ambient water samples collected from the subsurface and upper (300–500 m) and lower (1000 m) mesopelagic layer.

	Zooplankton samples				Water samples					
	Day		Night		100 m		300–500 m		1000 m	
Taxon	Average	SD	Average	SD	Average	SD	Average	SD	Average	SD
*Bacteroidetes_Flavobacteriia_Flavobacteriales*	32.3	11.8	33.7	8.2	16.0	8.3	9.5	5.7	8.8	5.8
*Proteobacteria_Alphaproteobacteria_Rhodobacterales*	23.8	10.6	20.9	6.8	5.0	3.2	1.3	1.0	2.1	3.6
*Proteobacteria_Betaproteobacteria_Burkholderiales*	10.7	16.8	9.1	14.0	0.0	0.0	0.0	0.0	0.0	0.0
*Proteobacteria_Gammaproteobacteria_ Pseudomonadales*	8.0	3.5	5.1	4.7	0.0	0.0	0.0	0.0	0.0	0.0
*Proteobacteria_Gammaproteobacteria_Alteromonadales*	2.4	1.0	3.6	2.6	3.1	1.5	2.7	1.4	2.9	1.6
*Proteobacteria_Gammaproteobacteria_Oceanospirillales*	0.3	0.5	1.1	2.5	7.8	1.7	7.0	3.3	6.4	2.3
*Proteobacteria_Alphaproteobacteria_Rickettsiales*	0.1	0.1	0.5	1.1	12.0	2.1	10.1	2.9	6.8	1.5
*Actinobacteria_Acidimicrobiia_Acidimicrobiales*	0.0	0.0	0.1	0.0	7.5	3.7	4.9	3.5	5.8	4.8
*Chloroflexi_*SAR202	0.0	0.0	0.0	0.0	3.1	5.8	5.5	5.1	6.4	5.0
P*roteobacteria_Deltaproteobacteria_*Sva0853	0.0	0.0	0.0	0.0	4.4	5.1	7.8	2.4	8.9	5.9
SAR406_Arctic96B‐7	0.0	0.0	0.0	0.0	3.1	1.5	4.2	1.5	4.8	1.4

### Ecotypes in the zooplankton‐associated bacterial community

**Figure 4:**
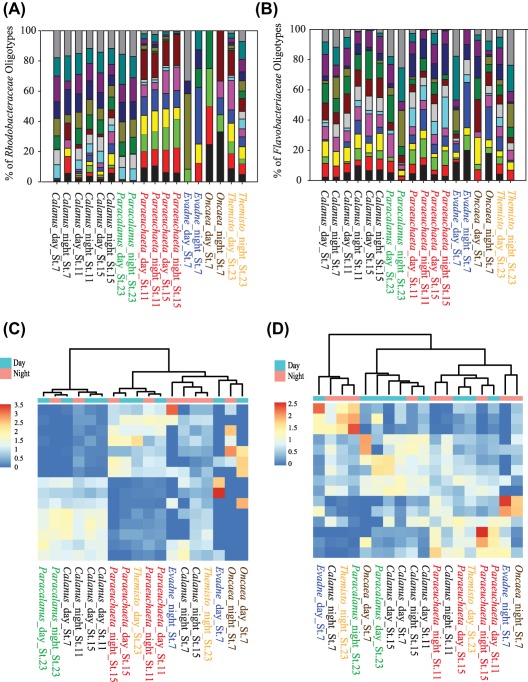
Contribution of *Rhodobacteraceae* (A) and *Flavobacteriaceae* (B) oligotypes obtained from different zooplankton species collected at specific stations and time of the day. Different colours represent specific oligotypes for each phylogenetic group. Heatmap showing the *z*‐score (numerical representation of a value's relationship to the mean of a group of values) distribution of *Rhodobacteraceae* (C) and *Flavobacteriaceae* (D) oligotypes among specific zooplankton species. The dendrogram clusters the samples according to the Bray Curtis similarity index.

Oligotyping, a supervised computational method to investigate the diversity of closely related but distinct bacterial organisms, was used to classify selected bacterial phylogenetic groups. Oligotyping analysis at the nucleotide level of the *Flavobacteriaceae* and *Rhodobacteraceae* (the two dominant families in the zooplankton‐associated bacterial community) identified 15 quality‐controlled oligotypes selected from the highest entropy values (using 16 and 53 components for *Flavobacteriaceae* and *Rhodobacteraceae* respectively) (Fig. 4A and B). The zooplankton‐associated oligotypes largely differed from the bacterioplankton oligotypes in the ambient water (Supporting Information Fig. S3). The *z*‐score distribution of the *Rhodobacteraceae* oligotypes resulted in two main clusters, in agreement with the clustering of samples obtained according to the Bray Curtis similarity index. The first cluster grouped samples obtained from *Calanus* sp. and *Paracalanus* sp., and the second one grouped samples obtained mainly from *Paraeuchaeta* sp. (Fig. 4A and C). *Flavobacteriaceae* oligotypes did not cluster according to zooplankton species, location or time of the day (Fig. 4B and D).

### Metagenomic analysis of the zooplankton‐associated microbial community

The metagenomic data obtained from the copepod‐associated (*Calanus* sp. and *Paraeuchaeta* sp.) microbial community were used to characterize the potential metabolic pathways present in the microbial consortium associated with the zooplankton's gut and/or carapace.

#### Genes indicative for membrane‐associated proteins

**Figure 5:**
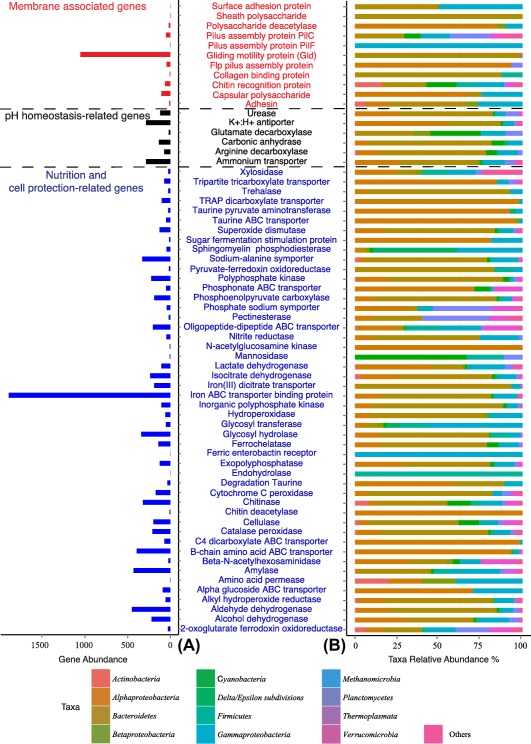
Number of reads (left panel) and their phylogenetic affiliation expressed in relative abundance (right panel) of genes associated to the main metabolic pathways obtained from the copepod‐associated bacterial communities (*Calanus* sp. and *Paraeuchaeata* sp.).

**Figure 6:**
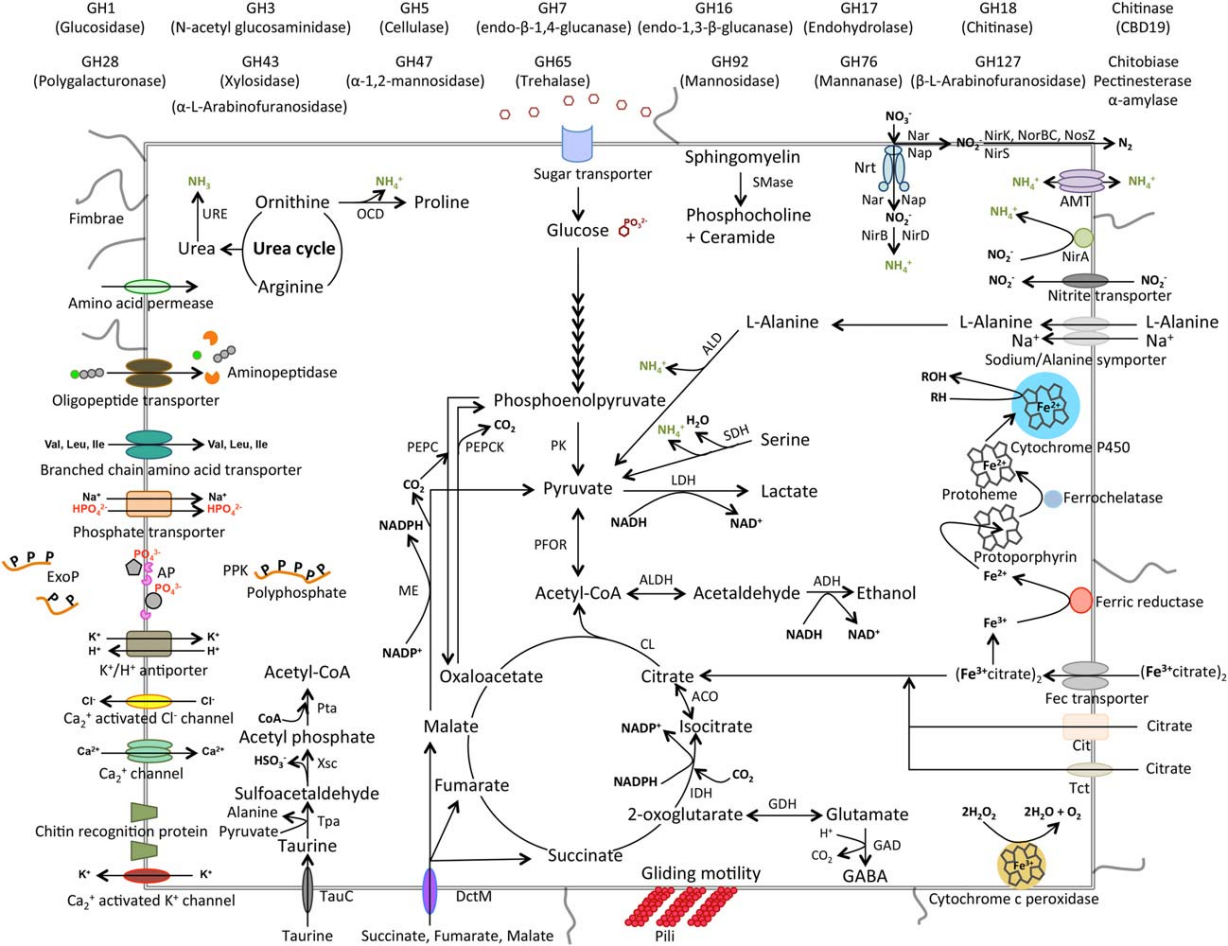
Metabolic interpretation of the copepod‐associated bacterial metagenome obtained from two copepod species (*Calanus* sp. and *Paraeuchaeata* sp.). Abbreviations are as follows: ACO, aconitase; ADH, alcohol dehydrogenase; ALD, alanine dehydrogenase; ALDH, aldehyde dehydrogenase; AMT, ammonium transporter; AP, alkaline phosphatase; CBD, carbohydrate binding domain; Cit, citrate transporter; CL, citrate lyase; Co‐A, Coenzyme‐A; Dct, C4‐dicarboxylate transport; ExoP, exopolyphosphatase; Fec, ferric dicitrate transport system; GABA, gamma aminobutyric acid; GAD, glutamate decarboxylase; GDH, glutamate dehydrogenase; GH, glycosyl hydrolase; IDH, isocitrate dehydrogenase; LDH, lactate dehydrogenase; ME, malic enzyme; NIR, nitrite reductase; Nrt, nitrate/nitrite transport system; Nar, nitrate reductase; Nap, periplasmic nitrate reductase; NirA, nitrite reductase (related to assimilatory nitrite reduction); NirB, NirD, nitrite reductase (associated to dissimilatory nitrite reduction); NirK, NirS, nitrite reductase (related to denitrification processes); Nor, Nitric oxide reductase (denitrification); Nos, nitrous‐oxide reductase (denitrification); OCD, ornithine cyclodeaminase; PEPC, phosphoenolpyruvate carboxylase; PEPCK, phosphoenolpyruvate carboxykinase; PFOR, pyruvate ferredoxin oxidoreductase; PK; pyruvate kinase; PPK, polyphosphate kinase; Pta, phosphate acetyltransferase; SDH, serine dehydratase; SMase, sphingomyelin phosphodiesterase; TauC, ABC‐type taurine transporter permease component; Tct, tricarobxylate transporter; Tpa, taurine‐pyruvate aminotransferase; URE, urease; Xsc, sulfoacetaldehyde acetyltransferase.

Several carbohydrate binding domain (CBD19) sequences such as chitinase and chitin recognition protein‐encoding genes were found in the metagenome (Figs 5 and 6). Genes from peptide binding domains encoding for proteins that facilitate binding of the cells to a saccharide‐based surface such as chitin (the main component of the zooplankton's carapace) were also abundant in the metagenomes from zooplankton‐associated bacterial communities (Fig. 5). Other genes involved in microbial adherence processes, such as pilus and fimbriae‐encoding genes, were also found in the zooplankton‐associated microbial community. Pilus protein‐encoding genes were mainly associated with *Gammaproteobacteria* (100% of the PilF genes and 17% of the PilC genes), *Bacteroidetes* (26% PilC genes), *Planctomycetes* (23% of the PilC genes), whereas, fimbriae protein‐encoding genes were affiliated to *Bacteroidetes* (27%) and *Planctomycete*s (23%) (Figs 5 and 6, Supporting Information Table S2). Genes involved in gliding motility were the most abundant membrane associated genes with 1053 assigned reads (*gld*A, *gld*D, *gld*F, *gld*G, *gld*H, *gld*I, *gld*J, *gld*K, *gld*L, *gld*M, *gld*N, *gld*O, *rem*B, *spr*A, *spr*B), predominantly associated to the *Bacteroidetes* phyla (98% of the total reads) (Fig. 5, Supporting Information Table S2).

#### Genes indicative of pH homeostasis

The metagenomic analysis indicated the presence of genes encoding proteins involved in cellular pH regulation mechanisms such as potassium/proton antiporter and proton conducting membrane transporters balancing [H^+^] ion concentrations within the cell to control acidity accounting for 289 reads (Figs 5 and 6, Supporting Information Table S2). These transporter‐associated genes were mainly affiliated with the *Alphaproteobacteria* (54%) and *Bacteroidetes* (32%) (Fig. 5, Supporting Information Table S2). The uptake of ammonium or release of ammonia may also be used to raise the cell's pH to control its cytoplasmic acidity. In this context, ammonia transporter and several metabolic genes, such as serine dehydratase (SDH), ornithine cyclodeaminase (OCD), alanine dehydrogenase (ALD) and dissimilatory nitrite reductase (NIRB, NIRD) were also found in the metagenome of zooplankton‐associated bacteria (Fig. 6, Supporting Information Table S2). The ammonium transporters accounted for a similar number of reads as the potassium/proton antiporter and proton transporters, mainly associated with *Alphaproteobacteria* (26%) and *Flavobacteriaceae* (47%), the two dominant groups of the zooplankton‐associated bacterial community (Fig. 5, Supporting Information Table S2). Additionally, several other genes, such as glutamate decarboxylase (GAD) (present in diverse phyla), arginine decarboxylase (mainly in *Bacteroidetes* 64%) and carbonic anhydrase (mainly associated with *Bacteroidetes* 72%) (Fig. 5, Supporting Information Table S2) may also play an important role in regulating the cellular pH by utilizing [H^+^] ions in their metabolic reactions and thus increasing the cytosol pH.

#### Energy transduction‐ and cell protection‐related genes

Several genes related to glycosyl hydrolase (GH), such as chitinase encoding genes (CBD19), were found in the metagenome (Fig. 6). Chitinase encoding genes were widespread among the bacterial community (Fig. 5) while chitin deacetylase was associated exclusively to *Bacteroidetes* (100% of the chitin deacetylase reads). Other genes involved in the degradation of complex molecules released by the zooplankton through digestive processes were also found. Cellulase accounted for 200 reads mainly associated with *Bacteroidetes* (53%), while amylase accounted for 431 reads mostly related to *Bacteroidetes* (42%) and *Gammaproteobacteria* (31%) (Fig. 5, Supporting Information Table S2). These two genes encode enzymes primarily involved in the degradation of cellulose, starch and other related polysaccharides of phytoplankton origin.

Additionally, metagenomic analysis revealed several genes encoding proteins involved in anaerobic metabolic pathways. Genes indicative of fermentative pathways were also present in the metagenomes, for example, genes encoding enzymes involved in the transformation of pyruvate into lactate [pyruvate ferredoxin oxidoreductase (PFOR)] and acetyl CoA into ethanol (aldehyde dehydrogenase, alcohol dehydrogenase and acetyl CoA synthase; ALDH, ADH) (Fig. 6), potentially generating oxidizing agents such as NAD^+^ for redox reactions (Fig. 6). Lactate dehydrogenase (LDH, Fig 6) and alcohol dehydrogenase (ALDH and ADH, Fig. 6) encoding genes were distributed amongst *Alphaproteobacteria*, *Gammaproteobacteria* and *Bacteroidetes* (Fig. 5).

Genes associated with denitrification and dissimilatory nitrate and nitrite reduction to ammonium (DNRA), such as *narG*, *narH*, *narI*, *napA*, *napB*, *nirB*, *nirD*, *nirK*, *nirS*, *norB*, *nosZ* (Fig.6, Supporting Information Table S2) were also found in the metagenome of the zooplankton‐associated microbial community. These genes were mainly associated with *Alphaproteobacteria*, *Gammaproteobacteria* and *Bacteroidetes*. Furthermore, a few genes usually associated to oxygen‐limited environments, such as PFOR (Fig. 6), citrate transporter (Cit, Tct), cytochrome c oxidase cbb3 and nitrite reductase (NIR, Fig. 6), were also detected in the metagenome. All these genes related to oxygen‐limited environments were mostly associated to *Bacteroidetes* (Fig. 5, Supporting Information Table S2). The zooplankton‐associated bacterial community also harboured genes involved in reductive TCA and anaplerotic carbon fixation pathways (isocitrate dehydrogenase, aconitase, citrate lyase; IDH, ACO, CL, Fig. 6), mainly associated with *Bacteroidetes* and *Alphaproteobacteria* groups [isocitrate dehydrogenase (IDH) genes, Fig. 5]. We also found genes encoding taurine‐pyruvate aminotransferase (Tpa) and sulfoacetaldehyde acetyltransferase (XsC) related to taurine metabolism and belonging mostly to *Alphaproteobacteria* (Figs 5 and 6), and genes indicative for cellular detoxification, such as laccase and cytochrome P_450_, peroxidase (Fig. 6). We also detected several genes involved in phosphorus utilization such as phosphate transporters, exopolyphosphatase (ExoP), alkaline phosphatase (AP) and polyphosphate kinase‐encoding (PPK) genes mainly associated to *Bacteroidetes* and *Alphaproteobacteria* (Figs 5 and 6, Supporting Information Table S2).

Additionally, the zooplankton‐associated bacterial community harboured many genes involved in iron utilization, primarily associated with *Bacteroidetes*, *Alphaproteobacteria* and *Gammaproteobacteria*. Iron ABC transporter and Fe^3+^‐dicitrate transporter *fecA* genes accounted for a large fraction of the iron related genes (with 200 and 1900 reads respectively, Fig. 5) whereas, iron chelation‐associated genes such as ferrochelatase gene were present only at moderate abundance (141 reads, Fig. 5). We have also detected a ferric reductase gene that encodes for an oxidoreductase to inter‐convert ferric (Fe^3+^) and ferrous (Fe^2+^) ion (Fig. 6).

## Discussion

### Bacteria‐mesozooplankton associations

Studies have shown that the bacterial community associated with crustacean zooplankton resides on the exoskeleton (epibiont) and/or is associated with the zooplankton's gut (endosymbiont) (Tang *et al*., 2010; Eckert and Pernthaler, 2014). Culture‐independent studies showed *Alphaproteobacteria* and *Actinobacteria* are the most abundant members of the bacterial community associated with marine and freshwater zooplankton, followed by *Bacilli* and *Gammaproteobacteria* (Grossart *et al*., 2009; De Corte *et al*., 2014). These previous findings are in partial agreement with those obtained in this study. We found that in the temperate and sub‐arctic North Atlantic Ocean, the zooplankton‐associated bacterial community is mainly composed of *Flavobacteria*, *Alphaproteobacteria* (particularly *Rhodobacterales*) and *Gammaproteobacteria* (Fig. 3). *Flavobacteria* represent the second most abundant clade after *Proteobacteria* in marine ecosystems (Glockner *et al*., 1999; Gomez‐Pereira *et al*., 2010). Members of this bacterial clade are able to degrade high molecular weight organic matter, such as cellulose and chitin, suggesting a commensal or parasitic interaction between *Flavobacteria* and zooplankton (Cottrell and Kirchman, 2000; Beier and Bertilsson, 2013). Zooplankton's moults and carcasses are also a major source of chitin in the ocean, and their colonization by bacteria may also play a key role the C and N cycling of the ocean (Tang *et al*., 2010). *Rhodobacteraceae*, the second most abundant family found in the zooplankton‐associated bacterial community (Fig. 3), have been reported to live associated with marine organisms, such as coral, sponges and microalgae (Ridley *et al*., 2005; Burke *et al*., 2011; Roder *et al*., 2014) and to contribute to biofilm formation (Pujalte *et al*., 2014), indicating that this group may play a major role in the colonization of the zooplankton exoskeleton.

The microbial community associated with the zooplankton's gut might consist of a transient (passing through the digestive system of the host) and a persistent bacterial community (Grossart *et al*., 2009; Tang *et al*., 2010). To test whether the diel cycle (which can be related to the feeding status) might influence the bacterial‐host interactions, zooplankton samples were collected at different times of the day (day vs. night). In contrast to a previous report (Grossart *et al*., 2010), our results do not indicate significant diel differences in the composition of the zooplankton‐associated bacterial community (Fig. 2). Therefore, the zooplankton‐associated bacterial community might have been shaped by other factors rather than the diel migration and/or feeding status. The taxa‐specific microbiome and the strong dependence on the sampling location (Fig. 2) suggest that the ambient water microbial community and the presence of a suitable host are likely the main factors determining the composition of the zooplankton‐associated bacterial community.

However, only a few 16S rRNA gene oligotypes within the specific bacterial taxa analysed (i.e., *Flavobacteriaceae*, *Rhodobacteraceae*) dominated the zooplankton‐associated communities. This suggests that the zooplankton‐associated bacterial community consists of specialized ecotypes belonging to only a few phylogenetic groups that act as an interactive community (such as a consortia) able to metabolize different compounds released by the zooplankton, either as exudates from the body surface or as a by‐product of the digestion processes occurring in the zooplankton gut.

### Zooplankton‐associated bacterial community and its implication in the global biogeochemical cycles

The zooplankton‐associated microbial community exploits zooplankton as a microhabitat. In this microhabitat, genes indicative for surface attachment and encoding for pili, fimbriae and chitin‐recognition proteins are used to colonize the zooplankton's internal and/or external surface (Tran *et al*., 2011; Bodelon *et al*., 2013).

The high number of glycosyl hydrolase encoding genes (mainly associated with the *Flavobacteria* clade, Fig. 5) suggests the capability of the zooplankton‐associated bacterial community to metabolize polysaccharides and amino‐sugars, such as cellulose or chitin respectively (Beier and Bertilsson, 2013). In agreement with our findings, *Flavobacteria* have been shown to be able to utilize chitin and *N*‐acetyl glucosamine (Cottrell and Kirchman, 2000). Taken together, these results suggest a tight association between crustacean zooplankton and *Flavobacteria*, the latter being able to metabolize high molecular weight organics from the zooplankton's exoskeleton. Intriguingly, we did not obtain sequences related to *Vibrio* spp., another important player in chitin mineralization often associated with crustacean zooplankton (Erken *et al*., 2015) in contrast to previous studies conducted in coastal systems (Montanari *et al*., 1999; Turner *et al*., 2009). This discrepancy could be explained by a relatively lower abundance of *Vibrio* ssp. in cold open ocean waters as compared to warm coastal regions (Vezzulli *et al*., 2012). Additionally, the presence of amylase and pectin esterase‐encoding genes suggests the capability of zooplankton‐associated bacteria to metabolize starch and pectin (Moal *et al*., 1987; Alderkamp *et al*., 2007) derived from crustacean zooplankton grazing on phytoplankton.

Metagenomic and ‐proteomic studies revealed that taurine might be an important substrate for heterotrophic marine bacteria (Poretsky *et al*., 2010; Sowell *et al*., 2011; Williams *et al*., 2012). The importance of taurine for bacterial growth has primarily been demonstrated using SAR11 cultures (Carini *et al*., 2013). The concentration and turnover rate of dissolved taurine in the ocean have only recently been determined (Clifford *et al*., 2017). Taurine is an organo‐sulfonate found in the tissues of marine invertebrates such as zooplankton and is a potential source of carbon, nitrogen and sulfur for heterotrophic bacteria (Williams *et al*., 2012; Carini *et al*., 2013). Thus, the copepod‐associated bacterial community is in close proximity to the primary source of taurine, the copepod's body. The presence of taurine catabolic genes in the metagenomes such as the taurine‐pyruvate aminotransferase and sulfo‐acetaldehyde acetyltransferase indicates the potential importance of taurine as a substrate for zooplankton‐associated bacterial communities (Figs 5 and 6). Surprisingly, even though most of the taurine catabolic genes were associated with *Alphaproteobacteria*, none were affiliated to SAR11, likely due to the low contribution of this clade to the zooplankton‐associated bacterial community.

The copepod's hindgut exhibits low oxygen concentrations (from suboxic to anoxic) and low pH (Tang *et al*., 2011) suggesting that copepods' guts are microhabitats suitable for anaerobic microbes able to tolerate acidic conditions. The metagenome of the zooplankton associated community harbours genes indicative for pH regulation of the cytosol's acidity by removing protons or using ammonia as proton scavenger to produce ammonium (Booth, 1985; Slonczewski *et al*., 2009).

Recent publications have documented not only high level of dissimilatory nitrate and nitrite reduction activity but also the presence of genes involved in DNRA pathways in marine zooplankton‐associated microbial communities (Glud *et al*., 2015; Stief *et al*., 2017). Genes encoding enzymes for DNRA were also retrieved in the metagenome presented here. However, our data (based on the relative gene abundances) point towards ammonium biosynthesis (thus, assimilatory nitrate/nitrite reduction) rather than N_2_ gas dissipation by bacteria (Supporting Information Table S2). In contrast to previous reports on the presence of anammox and anaerobic methane oxidation on zooplankton's carcasses sinking through oxygen minimum zone (Stief *et al*., 2017), the metagenome of zooplankton‐associated microbial communities from the open, well‐oxygenated Atlantic lacked genes indicative for these anaerobic pathways.

Microbes living at neutral or basic pH, such as marine free‐living bacteria, are exposed to low iron availability due to the insolubility of ferric iron (Fe^3+^). However, the environmental conditions in the copepod's gut, characterized by low pH and low oxygen (Tang *et al*., 2011), favour the bioavailable ferrous form (Fe^2+^) and thus have the potential to facilitate iron remineralization (Tang *et al*., 2011; Nuester *et al*., 2014; Schmidt *et al*., 2016). Therefore, ferrous ions could be directly available for the cytochrome c production without the need of ferric‐reductase (Schroder *et al*., 2003). However, the gene encoding this latter enzyme was relatively abundant in the copepod‐associated bacterial community (Fig. 5, Supporting Information Table S2), suggesting that iron is present in different forms in the zooplankton microhabitat. Thus, the zooplankton‐associated bacterial metabolic pathways could play an important role in the recycling of iron following the zooplankton grazing of diatoms in the euphotic layers in iron limited regions of the global ocean (Hutchins and Bruland, 1994; Hutchins *et al*., 1995). Moreover, the zooplankton grazing on phytoplankton may also play an important role in the phosphorus recycling of the open ocean (Corner, 1973; Olsen *et al*., 1986). The bioavailable phosphorus released by the zooplankton (mainly in form of inorganic phosphate) could be directly metabolized by the copepod‐associated bacterial community, which harbours large number of genes encoding for phosphate transporters (Fig. 5, Supporting Information Table S2). Hence, gut associated bacteria might scavenge phosphate within the gut and consequently reduce the number of phosphorous compounds released via faecal pellet production into the environment. Additionally, the presence of detoxification genes implies that the zooplankton‐associated bacterial community responds to the presence of toxic by‐products derived from digestive processes occurring in the host's gut.

The contribution of archaea to the zooplankton‐associated microbial community was negligible in the present study. Only a low number of reads associated to amylase of archaeal origin were found (Supporting Information Table S2). Our results suggest that in the zooplankton's gut, bacteria outcompete archaea. However, a previous study showed that zooplankton digestive tracts are most likely sites for methanogenesis (De Angelis and Lee, 1994), a process mediated by archaea. Our results further suggest that methane production by zooplankton‐associated communities is most likely a species‐specific process. Thus, only specific zooplankton species might be suitable hosts for methanogenic archaea and fuel methane production in oxygenated waters.

## Conclusion

In the North Atlantic Ocean, the bacterial community associated with crustacean zooplankton is mainly shaped by the zooplankton host (taxa‐specific interactions) and the bacterial community of the ambient water to which the zooplankton host is exposed. The zooplankton‐associated bacterial community is highly specialized, able to adhere and colonize internal and/or external surfaces, and to utilize high molecular weight organic compounds and metabolites, such as taurine released by zooplankton. Therefore, the zooplankton‐bacteria consortium can mediate specific biogeochemical processes (through the proliferation of specific bacterial taxa) that are generally underrepresented in the ambient waters. However, further studies to quantitatively assess the contribution of these communities to the global biogeochemical cycles are required.

## Experimental procedures

### Study area and sampling

Water samples were collected during the MEDEA‐II cruise (June–July 2012) at four different stations located between 50°51′N 28°51′W and 66°01′N 02°41′W (Supporting Information Fig. S4). Seawater samples were collected with a rosette sampler equipped with 25L Niskin bottles. To characterize the bacterial community of the ambient waters, 10 L of seawater were sampled from the lower euphotic layer (100 m) and the upper (300–500 m) and lower (1000 m) mesopelagic layer. The seawater was filtered onto 0.2 μm GTTP membrane filter (Millipore) and the filters stored at −80°C until further processing in the laboratory. Mesozooplankton samples were collected twice per day at the same station as the ambient water using vertical plankton tows (200 μm mesh size, hoisted at 30 m min^−1^) from 200 m during the night, and from 750 m depth during the day. These are the depth layers within which the majority of the crustaceous zooplankton migrates over a diel cycle (Steinberg *et al*., 2000; Tang *et al*., 2010). The content of the cod end of the plankton net was transferred into a plankton splitter and concentrated over a 70 μm mesh Nitex screen. The zooplankton samples were then transferred into 50 ml Greiner tubes and stored at −80°C until sorting. Once at the home laboratory, zooplankton individuals were thawed at room temperature and transferred to a Petri for sorting the dominant crustacean zooplankton taxa (i.e., *Calanus*, *Paracalanus*, *Paraeuchaeta*, *Themisto*, *Evadne* and *Oncaea*). To evaluate the zooplankton species‐associated bacterial community, 10 individuals of each taxon were collected under a dissecting microscope using clean forceps or a sterile pipette and transferred into sterile Eppendorf tubes for nucleic acid extraction.

### DNA extraction

The DNA of the ambient water samples was extracted using Ultraclean Soil DNA isolation Kit (MoBIO Laboratories). Zooplankton DNA was extracted using the phenol‐chloroform extraction protocol (Weinbauer *et al*., 2002), preceded by a bead‐beating step to facilitate lysis of the zooplankton individuals. NanoDrop ND‐1000 spectrophotometer (NanoDrop Technologies) was used to check the quality of the extracted DNA.

### Next generation sequencing and bioinformatics analyses of the bacterial 16S rRNA genes

The 16S rRNA genes of the zooplankton‐associated and ambient water bacterial communities were PCR amplified with the bacterial primers 341F (5′‐ CCTACGGGNGGCWGCAG‐3′) and 805R (5′‐GACTACHVGGGTATCTAATCC −3′) (Klindworth *et al*., 2013). PCR amplification of the 16S rRNA gene was carried out in 25 μl reaction volume using Fermentas Taq polymerase (Thermo Scientific) in a Mastercycler (Eppendorf) with the following parameters: initial denaturation at 95°C for 5 min, followed by 30 cycles of denaturation at 94°C for 1 min, annealing at 57.5°C for 30 s and extension at 72°C for 45 s, with a final extension at 72°C for 7 min. The PCR products were additionally purified with a PCR purification kit (5‐Prime). The quality of the PCR product was checked on 2% agarose gel. The 16S rDNA amplicons were subsequently sequenced with Illumina Miseq high‐throughput sequencing (2 × 250 bp paired‐end platform) at IMGM Laboratories GmbH (Martinsried, Germany).

The bioinformatic analysis of the 16S rRNA gene sequences followed the standard operating procedure pipeline of QIIME (Caporaso *et al*., 2010). Rarefaction curves, PD, Chao1, OTU richness, Shannon index of diversity and the Simpson evenness index were calculated with QIIME. Pairwise UniFrac distance and principal coordinate analysis (PCoA) (Lozupone *et al*., 2011) were used to compare the bacterial community composition between the samples (implemented in QIIME). A t‐test (implemented in Sigma Plot v.11) was used to assess differences between samples. Oligotyping analysis of Flavobacteriaceae and Rhodobacteraceae families was conducted following Eren's lab pipeline (available from http://oligotyping.org) (Eren *et al*., 2013).

### Prokaryotic DNA isolation, whole genome amplification and metagenomic analysis

Forty copepod individuals (20 *Calanus* sp. and 20 *Paraeuchaeate* sp.) were used for the metagenomic analysis of the zooplankton‐associated bacterial community. Since the genomic material extracted from the zooplankton‐associated samples contained both eukaryotic and prokaryotic DNA, DNA extracts were treated with the Looxster Enrichment kit (Analytikjena, Germany) following the manufacturer's protocol to enrich the bacterial DNA and remove the eukaryotic DNA. The resulting genetic material (enriched in prokaryotic DNA) was subsequently amplified with GenomePlex whole genome amplification kit (Sigma‐Aldrich) following the manufacturer's instructions. The quality of the amplified DNA was checked on 2% agarose, and the DNA was afterwards purified with a PCR purification kit (5‐Prime). The isolated DNA was used to construct a Nextera library (San Diego, USA). The obtained library was subsequently sequenced with Illumina Miseq high‐throughput sequencing (2 × 250bp paired‐end platform) at IMGM Laboratories GmbH (Martinsried, Germany).

The filtered prokaryotic reads obtained from the Illumina sequencing were screened for sequence similarity against KEGG GENES protein database using the DIAMOND BLASTX (Buchfink *et al*., 2015) with an e‐value cutoff of 10^−5^ and a minimum alignment length cutoff of 30 amino acids. Subsequently, the resulting reads were compared to NCBI non‐redundant database using DIAMOND BLASTX with default parameters. Taxonomic classification and functional annotation to KEGG functions was analysed using MEGAN v5.10 (Huson *et al*., 2007) with 50 LCA minimum score, 10^−5^ maximum expected and minimum support set to 1.

The sequence data generated are publicly available in the DDBJ database under the accession numbers DRA005574 (metagenome) and DRA005573 (amplicons).

## Availability of data and materials

The sequence data generated are publicly available in the DDBJ database under the accession numbers DRA005574 (metagenome) and DRA005573 (amplicons).

## Conflict of interest

The authors declare that they have no competing interests.

## Supporting information

Additional Supporting Information may be found in the online version of this article at the publisher's web‐site:

**Fig. S1.** Rarefaction curves (Phylogenetic Diversity [a], Chao index [b] and Observed OTUs [c]) obtained for the 16S rDNA sequences of zooplankton‐associated and ambient water bacterial communities. Operational taxonomic units (OTUs) were defined at 97% sequence identity.

**Fig. S2.** Venn diagram showing the shared and unique bacterial operational taxonomic units (OTUs) of zooplankton (Day and Night) and ambient water (Surface and Mesopelagic) samples expressed as relative contribution to the total number of OTUs (in %).

**Fig. S3.** Heatmap showing the *z*‐score distribution of *Rhodobacteraceae* (a) and *Flavobacteriaceae* (b) oligotypes of different zooplankton species and water samples collected at different stations.

**Fig. S4.** Sampling sites where mesozooplankton and water samples were collected (indicated by full circles) during the MEDEA II cruise in the North Atlantic.

**Table S1.** Total number of OTUs (cutoff 97% similarity), Chao species richness, phylogenetic and Shannon diversity indexes and Simpson evenness obtained from 16S rDNA sequences from ambient water and zooplankton‐associated bacteria.

**Table S2.** Number of reads for the main metabolic pathways of the *Calanus* sp, and *Paraeuchaeata* sp associated bacterial community, their phylogenetic affiliation (expressed in relative abundance) and their lowest and highest taxonomic identity (in %).
