# Supplementary figures and images for: Metagenomic insights into zooplankton‐associated bacterial communities
Source: Environ Microbiol. 2017 Oct 27;20(2):492–505. doi: 10.1111/1462-2920.13944 (PMC5836950; doi:10.1111/1462-2920.13944)

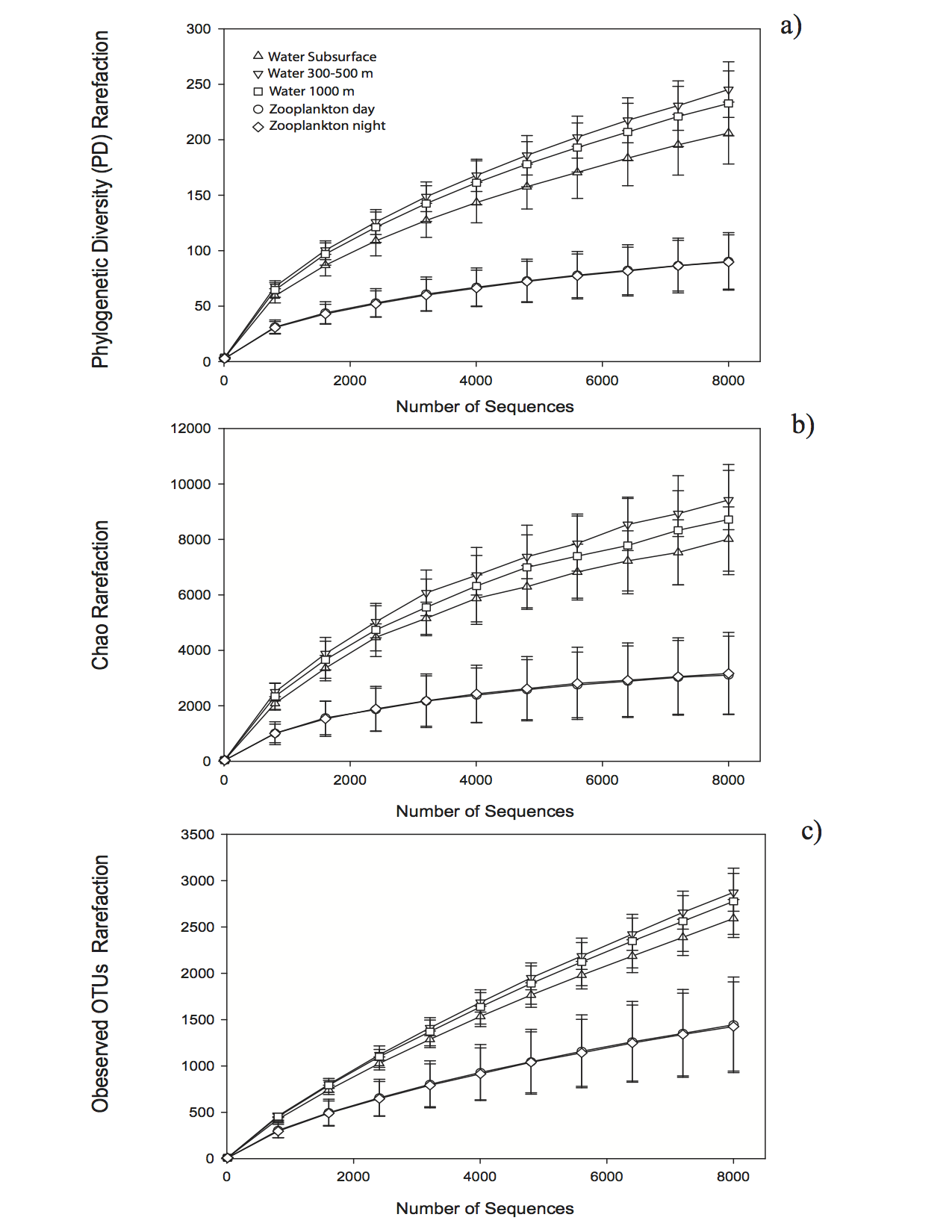

Supplement: Supplementary file 1 — Fig. S1. Rarefaction curves (Phylogenetic Diversity [a], Chao index [b] and Observed OTUs [c]) obtained for the 16S rDNA sequences of zooplankton‐associated and ambient water bacterial communities. Operational taxonomic units (OTUs) were defined at 97% sequence identity. [file EMI-20-492-s001.tiff]

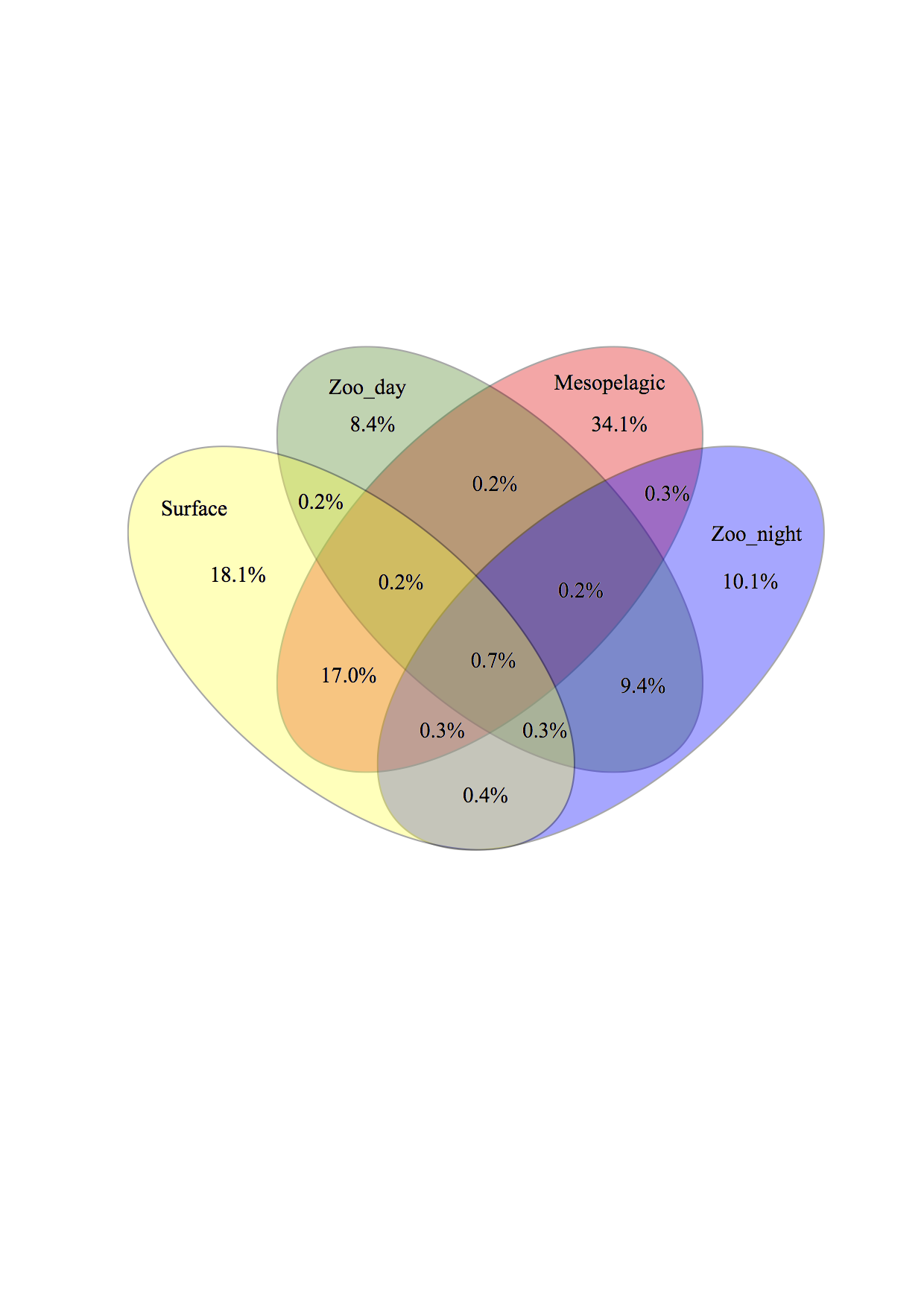

Supplement: Supplementary file 2 — Fig. S2. Venn diagram showing the shared and unique bacterial operational taxonomic units (OTUs) of zooplankton (Day and Night) and ambient water (Surface and Mesopelagic) samples expressed as relative contribution to the total number of OTUs (in %). [file EMI-20-492-s002.tiff]

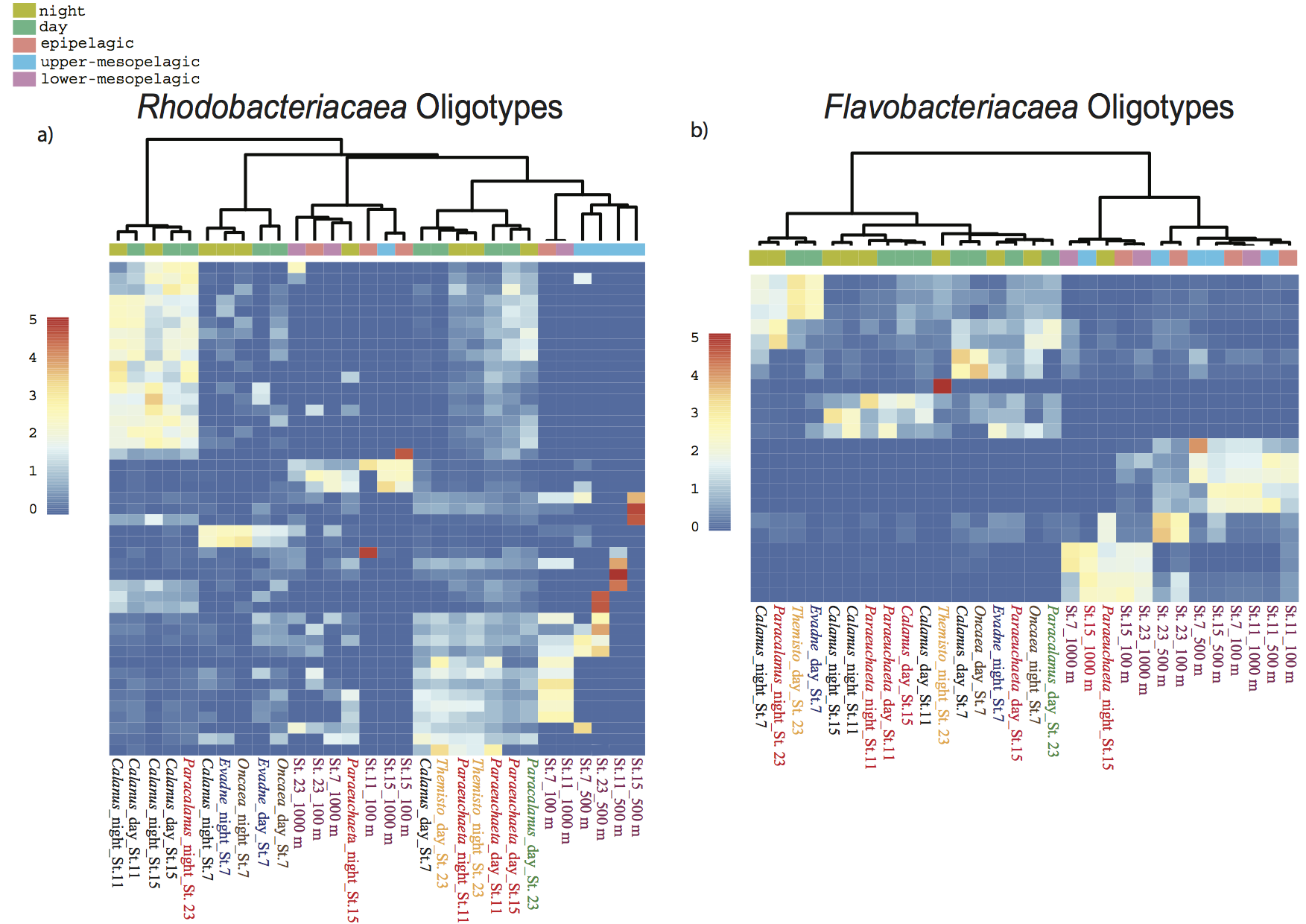

Supplement: Supplementary file 3 — Fig. S3. Heatmap showing the z‐score distribution of Rhodobacteraceae (a) and Flavobacteriaceae (b) oligotypes of different zooplankton species and water samples collected at different stations. [file EMI-20-492-s003.tiff]

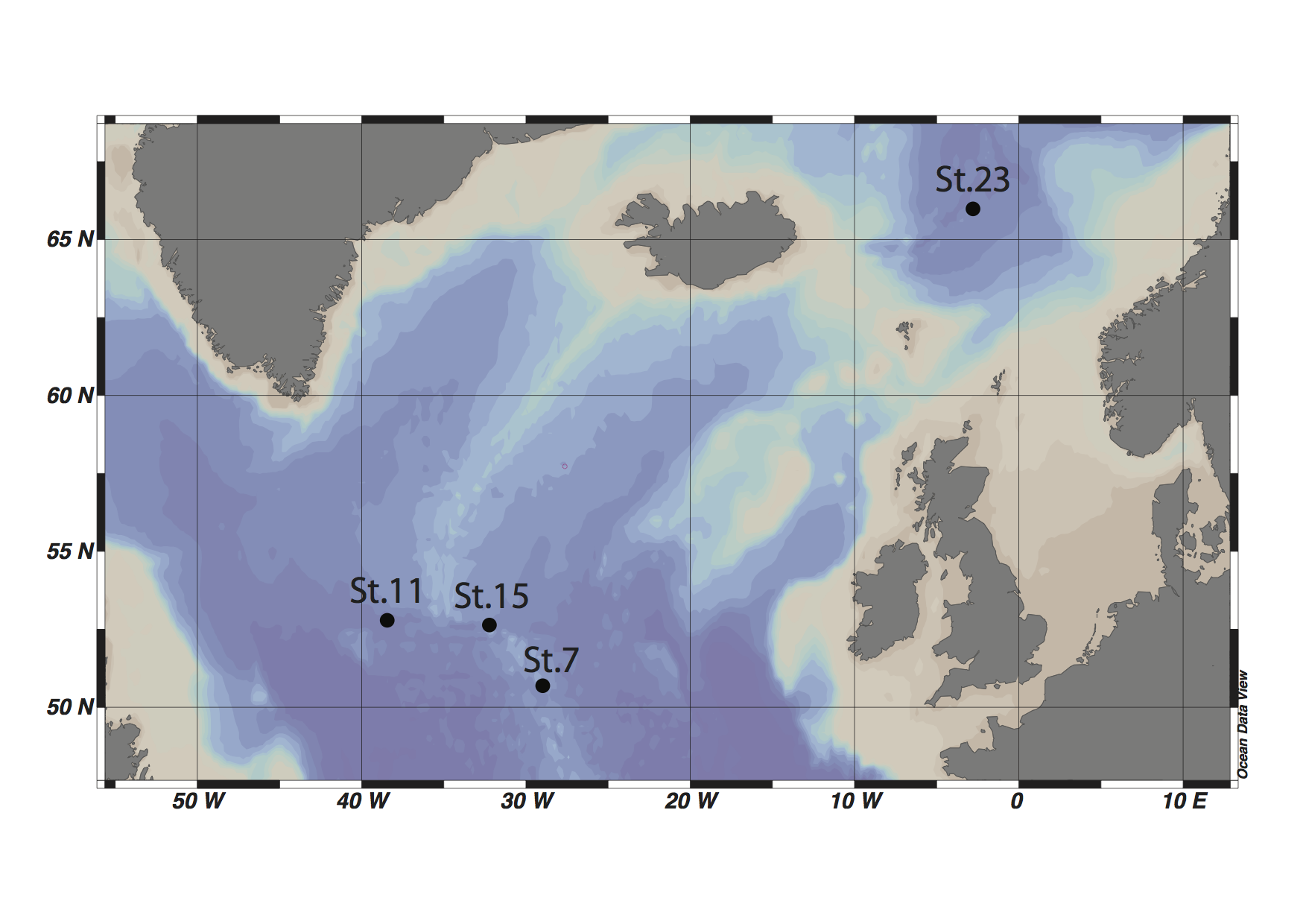

Supplement: Supplementary file 4 — Fig. S4. Sampling sites where mesozooplankton and water samples were collected (indicated by full circles) during the MEDEA II cruise in the North Atlantic. [file EMI-20-492-s004.tiff]
