# Supplementary material for: Metagenomic insights into zooplankton‐associated bacterial communities
Source: Environ Microbiol. 2017 Oct 27;20(2):492–505. doi: 10.1111/1462-2920.13944 (PMC5836950; doi:10.1111/1462-2920.13944)
Supplement: Supplementary file 5 — Table S1. Total number of OTUs (cutoff 97% similarity), Chao species richness, phylogenetic and Shannon diversity indexes and Simpson evenness obtained from 16S rDNA sequences from ambient water and zooplankton‐associated bacteria. [file EMI-20-492-s005.docx]

| Ambient Water | | | | | | | |
| --- | --- | --- | --- | --- | --- | --- | --- |
|  | Station | Depth (m) | OTUs observed | Chao | Phylogenetic Diversity | Shannon Diversity | Simpson Evenness |
|  |  |  |  |  |  |  |  |
|  | 7 | 100 | 12348 | 14126 | 671 | 9.9 | 0.005 |
|  | 7 | 500 | 11605 | 12082 | 710 | 10.4 | 0.007 |
|  | 7 | 1000 | 7885 | 8459 | 446 | 9.6 | 0.012 |
|  | 11 | 100 | 8446 | 9593 | 439 | 9.6 | 0.010 |
|  | 11 | 500 | 10509 | 12653 | 589 | 9.7 | 0.006 |
|  | 11 | 1000 | 12827 | 14235 | 686 | 10.1 | 0.005 |
|  | 15 | 100 | 9014 | 10276 | 501 | 9.9 | 0.010 |
|  | 15 | 300 | 9150 | 10919 | 517 | 9.8 | 0.010 |
|  | 15 | 1000 | 11773 | 13523 | 668 | 10.4 | 0.008 |
|  | 23 | 100 | 7533 | 8818 | 383 | 9.2 | 0.009 |
|  | 23 | 300 | 9960 | 11257 | 569 | 10.0 | 0.009 |
|  | 23 | 1000 | 5215 | 6601 | 354 | 9.1 | 0.010 |
|  |  |  |  |  |  |  |  |
| Zooplankton | | | | | | | |
| Species | Station | Depth (m) | OTUs observed | Chao | Phylogenetic Diversity | Shannon diversity | Simpson Evenness |
|  |  |  |  |  |  |  |  |
| *Calanus*_day | 7 | 250 | 2608 | 3188 | 145 | 8.4 | 0.028 |
| *Evadne_*day | 7 | 250 | 870 | 1091 | 56 | 5.6 | 0.015 |
| *Oncaea_*day | 7 | 250 | 1113 | 1418 | 71 | 5.5 | 0.009 |
| *Evadne_*night | 7 | 750 | 808 | 1256 | 59 | 5.9 | 0.017 |
| *Oncaea*_night | 7 | 750 | 1355 | 1877 | 85 | 7.0 | 0.021 |
| *Calanus_*night | 7 | 750 | 1574 | 2157 | 95 | 7.9 | 0.039 |
| *Paraeuchaeta*_day | 11 | 250 | 5171 | 6465 | 218 | 9.0 | 0.019 |
| *Calanus_*day | 11 | 250 | 4639 | 5762 | 201 | 8.8 | 0.017 |
| *Paraeuchaeta_*night | 11 | 750 | 3905 | 4833 | 175 | 8.1 | 0.012 |
| *Calanus_*night | 11 | 750 | 3239 | 4071 | 145 | 7.5 | 0.013 |
| *Paraeuchaeta_*day | 15 | 250 | 3823 | 4798 | 183 | 9.2 | 0.035 |
| *Calanus_*day | 15 | 250 | 3093 | 4147 | 158 | 8.4 | 0.019 |
| *Paraeuchaeta*_night | 15 | 750 | 6878 | 7865 | 280 | 9.6 | 0.019 |
| *Calanus*_night | 15 | 750 | 6256 | 7340 | 278 | 9.5 | 0.025 |
| *Themisto*_day | 23 | 250 | 5427 | 6393 | 227 | 8.7 | 0.016 |
| *Paracalanus_*day | 23 | 250 | 984 | 1463 | 69 | 6.7 | 0.028 |
| *Themisto*_night | 23 | 750 | 3379 | 3758 | 167 | 6.7 | 0.005 |
| *Paracalanus*_night | 23 | 750 | 4922 | 5437 | 214 | 7.6 | 0.007 |

Table S1. Total number of OTUs (cutoff 97% similarity), Chao species richness, phylogenetic and Shannon diversity indexes and Simpson evenness obtained from 16S rDNA sequences from ambient water and zooplankton-associated bacteria.
