# Supplementary material for: Metagenomic insights into zooplankton‐associated bacterial communities
Source: Environ Microbiol. 2017 Oct 27;20(2):492–505. doi: 10.1111/1462-2920.13944 (PMC5836950; doi:10.1111/1462-2920.13944)
Supplement: Supplementary file 6 — Table S2. Number of reads for the main metabolic pathways of the Calanus sp, and Paraeuchaeata sp associated bacterial community, their phylogenetic affiliation (expressed in relative abundance) and their lowest and highest taxonomic identity (in %). [file EMI-20-492-s006.docx]

|  |  |  |  |  |  |  |  |  |  |  |  |  |  |  |  |  |  |  |
| --- | --- | --- | --- | --- | --- | --- | --- | --- | --- | --- | --- | --- | --- | --- | --- | --- | --- | --- |
|  | **Predicted gene function** | **Actinobacteria** | **Alphaproteobacteria** | **Bacteroidetes** | **Betaproteobacteria** | **Cyanobacteria** | **Delta/Epsilon subdivisions** | **Firmicutes** | **Gammaproteobacteria** | **Methanomicrobia** | **Planctomycetes** | **Thermoplasmata** | **Verrucomicrobia** | **Others** | **Number of reads** | **Lowest_ID** | **Highest_ID** | **Gene product** |
| **Surface association** | Surface adhesion protein | 0.0 | 0.0 | 50.0 | 0.0 | 0.0 | 0.0 | 0.0 | 50.0 | 0.0 | 0.0 | 0.0 | 0.0 | 0.0 | 2 | 41 | 47 | surface adhesion protein |
|  | Sheath polysaccharide | 0.0 | 0.0 | 100.0 | 0.0 | 0.0 | 0.0 | 0.0 | 0.0 | 0.0 | 0.0 | 0.0 | 0.0 | 0.0 | 4 | 71 | 75 | sheath polysaccharide |
|  | Polysaccharide deacetylase | 0.0 | 85.0 | 0.0 | 5.0 | 0.0 | 0.0 | 0.0 | 10.0 | 0.0 | 0.0 | 0.0 | 0.0 | 0.0 | 20 | 40 | 100 | polysaccharide deacetylase |
|  | Pilus assembly protein PilC | 0.0 | 2.0 | 27.5 | 0.0 | 9.8 | 0.0 | 0.0 | 17.6 | 0.0 | 23.5 | 0.0 | 7.8 | 11.8 | 51 | 31 | 100 | type IV fimbrial assembly protein PilC |
|  | Pilus assembly protein PilF | 0.0 | 0.0 | 0.0 | 0.0 | 0.0 | 0.0 | 0.0 | 100.0 | 0.0 | 0.0 | 0.0 | 0.0 | 0.0 | 4 | 51 | 91 | type IV pilus assembly protein PilF |
|  | Gliding motility protein (Gld) | 0.0 | 0.2 | 98.8 | 0.0 | 0.2 | 0.0 | 0.0 | 0.0 | 0.0 | 0.0 | 0.0 | 0.0 | 0.9 | 1053 | 39 | 100 | Gliding motility protein (GldA, B, C, D, E, F, G, H, I, J, K, L, M, N, O, RemB, SprA, B) |
|  | Flp pilus assembly protein | 0.0 | 93.3 | 0.0 | 0.0 | 0.0 | 0.0 | 0.0 | 0.0 | 0.0 | 6.7 | 0.0 | 0.0 | 0.0 | 45 | 51 | 90 | tight adherence protein |
|  | Collagen binding protein | 0.0 | 0.0 | 87.5 | 0.0 | 0.0 | 0.0 | 12.5 | 0.0 | 0.0 | 0.0 | 0.0 | 0.0 | 0.0 | 8 | 30 | 100 | collagen_binding_protein |
|  | Chitin recognition protein | 15.9 | 1.6 | 23.8 | 1.6 | 17.5 | 1.6 | 9.5 | 15.9 | 1.6 | 1.6 | 0.0 | 4.8 | 4.8 | 63 | 28 | 88 | chitinase |
|  | Capsular polysaccharide | 0.0 | 67.9 | 7.5 | 0.0 | 0.0 | 0.0 | 0.0 | 24.5 | 0.0 | 0.0 | 0.0 | 0.0 | 0.0 | 53 | 32 | 100 | capsular polysaccharide export protein |
|  | Adhesin | 6.7 | 13.3 | 46.7 | 6.7 | 0.0 | 0.0 | 0.0 | 26.7 | 0.0 | 0.0 | 0.0 | 0.0 | 0.0 | 15 | 32 | 83 | adhesin |
| **PH homeostasis** | Urease | 0.0 | 25.8 | 55.8 | 0.8 | 0.8 | 0.0 | 0.8 | 5.8 | 0.0 | 7.5 | 0.0 | 2.5 | 0.0 | 120 | 64 | 100 | urease |
|  | K^+^:H^+^ antiporter | 0.0 | 54.4 | 32.6 | 0.0 | 1.1 | 0.4 | 0.7 | 5.6 | 0.0 | 1.8 | 0.0 | 2.5 | 1.0 | 289 | 39 | 100 | multicomponent K+:H+ antiporter |
|  | Carbonic anhydrase | 0.0 | 8.9 | 72.6 | 0.0 | 7.4 | 2.2 | 0.7 | 8.1 | 0.0 | 0.0 | 0.0 | 0.0 | 0.0 | 156 | 37 | 100 | carbonic anhydrase |
|  | Ammonium transporter | 0.0 | 26.0 | 47.0 | 2.0 | 1.0 | 1.0 | 1.0 | 12.0 | 0.0 | 6.0 | 0.0 | 2.0 | 2.0 | 286 | 39 | 100 | ammonium transporter, Amt family |
|  | Arginine decarboxylase | 1.4 | 12.3 | 64.4 | 0.0 | 6.8 | 0.0 | 0.0 | 12.3 | 0.0 | 2.7 | 0.0 | 0.0 | 0.0 | 74 | 52 | 100 | arginine decarboxylase |
|  | Glutamate decarboxylase | 0.0 | 0.0 | 35.0 | 10.0 | 30.0 | 0.0 | 0.0 | 15.0 | 0.0 | 10.0 | 0.0 | 0.0 | 0.0 | 31 | 41 | 100 | glutamate decarboxylase |
| **Nutrition and cell protection-related** | Xylosidase | 0.0 | 24.0 | 12.0 | 4.0 | 0.0 | 0.0 | 16.0 | 16.0 | 0.0 | 4.0 | 0.0 | 0.0 | 24.0 | 25 | 41 | 84 | xylan 1,4-beta-xylosidase |
|  | Trehalase | 0.0 | 7.7 | 84.6 | 0.0 | 0.0 | 0.0 | 0.0 | 7.7 | 0.0 | 0.0 | 0.0 | 0.0 | 0.0 | 26 | 36 | 100 | alpha,alpha-trehalase |
|  | Taurine pyruvate aminotransferase | 0.0 | 92.3 | 0.0 | 0.0 | 0.0 | 3.8 | 0.0 | 3.8 | 0.0 | 0.0 | 0.0 | 0.0 | 0.0 | 26 | 68 | 96 | taurine-pyruvate aminotransferase |
|  | Taurine ABC transporter | 0.0 | 94.2 | 1.9 | 1.9 | 0.0 | 0.0 | 0.0 | 1.9 | 0.0 | 0.0 | 0.0 | 0.0 | 0.0 | 52 | 41 | 100 | taurine transport system ATP-binding protein/permease protein |
|  | Tripartite tricarboxylate transporter | 0.0 | 84.7 | 0.0 | 0.0 | 0.0 | 0.0 | 0.0 | 6.9 | 0.0 | 4.2 | 0.0 | 2.8 | 1.4 | 72 | 45 | 100 | tricarboxylic transport membrane protein |
|  | TRAP dicarboxylate transporter | 0.0 | 96.8 | 0.0 | 0.0 | 0.0 | 0.0 | 3.2 | 0.0 | 0.0 | 0.0 | 0.0 | 0.0 | 0.0 | 31 | 53 | 94 | C_4_-dicarboxylate transporter |
|  | Superoxide dismutase | 0.0 | 18.1 | 65.4 | 0.0 | 1.6 | 0.8 | 2.4 | 6.3 | 0.0 | 1.6 | 0.0 | 1.6 | 2.4 | 127 | 40 | 100 | superoxide dismutase, Fe-Mn family/ Cu-Zn family |
|  | Sugar fermentation stimulation protein | 0.0 | 81.3 | 0.0 | 0.0 | 0.0 | 0.0 | 0.0 | 18.8 | 0.0 | 0.0 | 0.0 | 0.0 | 0.0 | 16 | 59 | 87 | sugar fermentation stimulation protein A |
|  | Sphingomyelin phosphodiesterase | 0.0 | 4.3 | 2.2 | 2.2 | 2.2 | 50.0 | 0.0 | 39.1 | 0.0 | 0.0 | 0.0 | 0.0 | 0.0 | 46 | 31 | 72 | sphingomyelin phosphodiesterase |
|  | Sodium-alanine symporter | 2.1 | 6.1 | 70.3 | 0.3 | 1.8 | 0.0 | 1.2 | 15.5 | 0.0 | 0.3 | 0.0 | 2.4 | 0.0 | 330 | 38 | 100 | alanine or glycine:cation symporter, AGCS family |
|  | Pyruvate-ferredoxin oxidoreductase | 0.0 | 0.0 | 83.3 | 0.0 | 0.0 | 0.0 | 11.1 | 5.6 | 0.0 | 0.0 | 0.0 | 0.0 | 0.0 | 18 | 68 | 100 | pyruvate-ferredoxin oxidoreductase |
|  | Phosphoenolpyruvate carboxylase | 0.0 | 11.1 | 73.0 | 0.5 | 1.1 | 0.5 | 2.1 | 5.3 | 0.0 | 0.0 | 0.0 | 4.2 | 2.1 | 189 | 39 | 100 | polyphosphate kinase |
|  | Pectinesterase | 0.0 | 10.0 | 30.0 | 0.0 | 0.0 | 0.0 | 0.0 | 0.0 | 0.0 | 40.0 | 0.0 | 10.0 | 10.0 | 20 | 39 | 62 | pectinesterase |
|  | Oligopeptide-dipeptide ABC transporter | 1.0 | 24.3 | 3.9 | 0.0 | 0.5 | 2.4 | 18.4 | 24.8 | 0.0 | 1.0 | 0.0 | 22.3 | 1.5 | 206 | 44 | 99 | oligopeptide transport system permease protein |
|  | Assimilatory nitrate reduction | 0.0 | 9.7 | 31.9 | 0.0 | 16.7 | 0.0 | 0.0 | 12.5 | 0.0 | 4.2 | 0.0 | 15.3 | 9.7 | 72 | 46 | 100 | nitrite reductase |
|  | Dissimilatory nitrate reduction | 0.0 | 17.3 | 57.7 | 0.0 | 0.0 | 0.0 | 0.0 | 23.1 | 0.0 | 1.9 | 0.0 | 0.0 | 0.0 | 52 | 37 | 97 | nitrite reductase |
|  | Nitrate/nitrite transport system | 0.0 | 17.9 | 42.9 | 0.0 | 0.0 | 0.0 | 0.0 | 14.3 | 0.0 | 1.8 | 0.0 | 21.1 | 1.8 | 56 | 60 | 100 | nitrate/nitrite transport system |
|  | Nitrate/nitrite reductase (Denitrification) | 5.9 | 35.3 | 29.4 | 0.0 | 0.0 | 0.0 | 5.9 | 17.6 | 0.0 | 2.1 | 0.0 | 5.9 | 0.0 | 17 | 66 | 100 | nitrite reductase |
|  | Nitric /Nitrous-oxide reductase (Denitrification) | 0.0 | 23.5 | 52.9 | 0.0 | 0.0 | 0.0 | 0.0 | 17.6 | 0.0 | 0.0 | 0.0 | 0.0 | 5.9 | 17 | 66 | 100 | nitric /nitrous-oxide reductase |
|  | N-acetylglucosamine kinase | 0.0 | 100.0 | 0.0 | 0.0 | 0.0 | 0.0 | 0.0 | 0.0 | 0.0 | 0.0 | 0.0 | 0.0 | 0.0 | 8 | 42 | 65 | N-acetylglucosamine kinase |
|  | Mannosidase | 0.0 | 0.0 | 0.0 | 0.0 | 66.7 | 0.0 | 22.2 | 0.0 | 0.0 | 11.1 | 0.0 | 0.0 | 0.0 | 9 | 32 | 100 | alpha-amylase/alpha-mannosidase |
|  | Isocitrate dehydrogenase | 3.8 | 21.5 | 56.5 | 0.0 | 1.7 | 1.3 | 2.1 | 9.3 | 0.0 | 1.7 | 0.0 | 1.7 | 0.4 | 237 | 30 | 100 | isocitrate dehydrogenase |
|  | Iron(III) dicitrate transporter | 0.0 | 1.1 | 92.1 | 0.0 | 0.0 | 0.0 | 0.0 | 5.8 | 0.0 | 0.0 | 0.0 | 0.5 | 0.5 | 189 | 35 | 100 | Fe^3+^ dicitrate transport protein |
|  | Iron ABC transporter substrate binding protein | 0.5 | 14.7 | 66.1 | 0.3 | 0.9 | 0.1 | 1.1 | 13.4 | 0.0 | 0.2 | 0.0 | 0.9 | 2.0 | 1890 | 40 | 100 | iron complex transport system ATP-binding protein |
|  | Glycosyl transferase | 0.0 | 5.7 | 7.5 | 3.8 | 1.9 | 7.5 | 17.0 | 54.7 | 1.9 | 0.0 | 0.0 | 0.0 | 0.0 | 53 | 28 | 84 | glycosyl transferase |
|  | Glycosyl hydrolase | 0.9 | 12.3 | 66.6 | 0.6 | 0.6 | 0.0 | 10.9 | 6.5 | 0.6 | 0.3 | 0.0 | 0.9 | 0.0 | 341 | 23 | 100 | glucosidase/glucosylceramidase /glycanase |
|  | Ferrochelatase | 0.0 | 14.9 | 63.8 | 0.0 | 7.1 | 0.0 | 2.1 | 9.9 | 0.0 | 1.4 | 0.0 | 0.7 | 0.0 | 141 | 45 | 100 | ferrochelatase |
|  | Ferric enterobactin receptor | 0.0 | 0.0 | 0.0 | 0.0 | 0.0 | 0.0 | 0.0 | 100.0 | 0.0 | 0.0 | 0.0 | 0.0 | 0.0 | 2 | 33 | 92 | ferric enterobactin receptor |
|  | Exopolyphosphatase | 0.0 | 29.4 | 50.8 | 0.8 | 1.6 | 0.8 | 0.8 | 11.1 | 0.0 | 1.6 | 0.0 | 3.2 | 0.0 | 126 | 36 | 100 | exopolyphosphatase / guanosine-5'-triphosphate,3'-diphosphate pyrophosphatase |
|  | Endohydrolase | 0.0 | 0.0 | 0.0 | 0.0 | 0.0 | 0.0 | 100.0 | 0.0 | 0.0 | 0.0 | 0.0 | 0.0 | 0.0 | 3 | 39 | 58 | glucan endo-1,3-beta-D-glucosidase |
|  | Taurine degradation gene | 0.0 | 58.3 | 30.6 | 0.0 | 0.0 | 0.0 | 2.8 | 8.3 | 0.0 | 0.0 | 0.0 | 0.0 | 0.0 | 36 | 36 | 100 | GntR family transcriptional regulator / MocR family aminotransferase |
|  | Cytochrome C peroxidase | 0.0 | 7.6 | 74.4 | 0.0 | 0.0 | 0.0 | 0.0 | 5.8 | 0.0 | 5.2 | 0.0 | 3.5 | 3.5 | 172 | 36 | 100 | cytochrome c peroxidase |
|  | Cellulase | 2.5 | 5.5 | 53.0 | 1.0 | 12.0 | 0.0 | 4.5 | 6.0 | 1.0 | 3.0 | 0.0 | 9.0 | 2.5 | 200 | 26 | 100 | endoglucanase |
|  | C_4_-dicarboxylate ABC transporter | 0.0 | 98.6 | 0.0 | 0.0 | 0.0 | 0.0 | 0.0 | 1.4 | 0.0 | 0.0 | 0.0 | 0.0 | 0.0 | 70 | 53 | 94 | C4-dicarboxylate transporter |
|  | Branched-chain amino acid ABC transporter | 0.3 | 92.4 | 0.3 | 0.5 | 0.0 | 1.0 | 1.8 | 1.8 | 0.0 | 0.8 | 0.0 | 0.3 | 1.0 | 395 | 41 | 100 | branched-chain amino acid transport system |
|  | Amylase | 0.7 | 0.9 | 42.0 | 1.9 | 1.2 | 1.4 | 7.7 | 30.9 | 0.0 | 2.3 | 7.0 | 0.0 | 4.2 | 431 | 30 | 100 | alpha-amylase |
|  | Amino acid permease | 20.0 | 20.0 | 0.0 | 20.0 | 0.0 | 0.0 | 0.0 | 40.0 | 0.0 | 0.0 | 0.0 | 0.0 | 0.0 | 5 | 35 | 100 | solute carrier family |
|  | Alpha glucoside ABC transporter | 0.0 | 70.0 | 0.0 | 0.0 | 0.0 | 0.0 | 0.0 | 30.0 | 0.0 | 0.0 | 0.0 | 0.0 | 0.0 | 90 | 25 | 100 | alpha-glucoside transport system substrate-binding protein |
|  | Alcohol dehydrogenase | 0.0 | 0.0 | 0.0 | 0.0 | 0.0 | 0.0 | 66.7 | 33.3 | 0.0 | 0.0 | 0.0 | 0.0 | 0.0 | 3 | 96 | 100 | acetaldehyde/ alcohol dehydrogenase |
|  | Polyphosphate kinase | 0.9 | 18.2 | 68.4 | 0.9 | 3.6 | 0.4 | 0.4 | 1.8 | 0.0 | 1.8 | 0.0 | 3.1 | 0.4 | 225 | 43 | 100 | phosphonate transport system ATP-binding protein/permease |
|  | Phosphonate ABC transporter | 0.0 | 71.2 | 0.0 | 0.0 | 9.6 | 0.0 | 0.0 | 1.9 | 0.0 | 0.0 | 0.0 | 17.3 | 0.0 | 52 | 34 | 100 | phosphoenolpyruvate carboxylase |
|  | Phosphate sodium symporter | 0.0 | 30.2 | 7.0 | 0.0 | 0.0 | 0.0 | 2.3 | 7.0 | 0.0 | 34.9 | 0.0 | 16.3 | 2.3 | 43 | 33 | 98 | phosphate:Na^+^ symporter |
|  | Lactate dehydrogenase | 1.0 | 44.8 | 18.1 | 1.9 | 1.0 | 0.0 | 1.9 | 21.0 | 0.0 | 4.8 | 0.0 | 0.0 | 5.7 | 105 | 36 | 94 | L/D-lactate dehydrogenase |
|  | Iron Alcohol dehydrogenase | 0.5 | 30.4 | 40.1 | 0.9 | 1.4 | 0.5 | 1.8 | 16.1 | 0.0 | 6.0 | 0.0 | 0.5 | 1.8 | 217 | 43 | 100 | alcohol dehydrogenase |
|  | Inorganic polyphosphate kinase | 0.0 | 10.5 | 77.1 | 1.0 | 1.9 | 0.0 | 0.0 | 6.7 | 0.0 | 1.9 | 0.0 | 0.0 | 1.0 | 105 | 41 | 100 | NAD+ kinase |
|  | Chitinase | 8.1 | 4.0 | 41.9 | 1.2 | 13.7 | 0.6 | 7.1 | 9.9 | 1.2 | 2.2 | 0.0 | 7.1 | 2.8 | 322 | 28 | 88 | chitinase |
|  | Chitin deacetylase | 0.0 | 100.0 | 0.0 | 0.0 | 0.0 | 0.0 | 0.0 | 0.0 | 0.0 | 0.0 | 0.0 | 0.0 | 0.0 | 11 | 59 | 84 | chitin deacetylase |
|  | Beta-N-acetylhexosaminidase | 0.0 | 33.3 | 20.8 | 4.2 | 4.2 | 0.0 | 0.0 | 12.5 | 0.0 | 0.0 | 0.0 | 20.8 | 4.2 | 24 | 33 | 93 | beta-N-acetylhexosaminidase |
|  | Aldehyde dehydrogenase | 1.3 | 20.4 | 61.8 | 1.1 | 1.3 | 0.0 | 2.0 | 6.2 | 0.0 | 3.6 | 0.0 | 1.1 | 1.1 | 450 | 40 | 100 | NADP-dependent aldehyde dehydrogenase |
|  | 2-oxoglutarate ferrodoxin oxidoreductase | 6.7 | 10.0 | 23.3 | 0.0 | 0.0 | 0.0 | 3.3 | 16.7 | 0.0 | 20.0 | 0.0 | 13.3 | 6.7 | 30 | 50 | 100 | 2-oxoglutarate/2-oxoacid ferredoxin oxidoreductase |
|  | Hydroperoxidase | 0.0 | 8.5 | 67.8 | 3.4 | 0.0 | 0.0 | 3.4 | 16.9 | 0.0 | 0.0 | 0.0 | 0.0 | 0.0 | 59 | 62 | 100 | catalase |
|  | Catalase peroxidase | 1.4 | 13.7 | 64.2 | 0.5 | 0.9 | 0.5 | 2.4 | 9.0 | 0.0 | 2.8 | 0.0 | 0.9 | 3.8 | 212 | 38 | 100 | catalase-peroxidase |
|  | Alkyl hydroperoxide reductase | 1.7 | 15.5 | 63.8 | 1.7 | 0.0 | 0.0 | 1.7 | 10.3 | 0.0 | 1.7 | 0.0 | 0.0 | 3.4 | 58 | 44 | 100 | alkyl hydroperoxide reductase |

Table S2. Number of reads for the main metabolic pathways of the *Calanus* sp*,* and *Paraeuchaeata* sp associated bacterial community, their phylogenetic affiliation (expressed in relative abundance) and their lowest and highest taxonomic identity (in %)
